# Unraveling the roles of *IFIT3* gene and immune-metabolic pathways in psoriasis: a bioinformatics exploration for diagnostic markers and therapeutic targets

**DOI:** 10.3389/fmolb.2024.1439837

**Published:** 2024-08-22

**Authors:** Guangshan Chen, Xi Chen, Xingwu Duan, Runtian Zhang, Chunxiao Bai

**Affiliations:** ^1^ Department of Dermatology, Dongzhimen Hospital, Beijing University of Chinese Medicine, Beijing, China; ^2^ Department of Orthopedics, Dongzhimen Hospital, Beijing University of Chinese Medicine, Beijing, China

**Keywords:** psoriasis, *IFIT3*, differentially expressed genes, receiver operating characteristics (ROC) analysis, protein–protein network analysis

## Abstract

**Background:**

The functions and related signal pathways of the *IFIT3* gene in the skin lesions of patients with psoriasis were explored through bioinformatics methods to determine the potential specific molecular markers of psoriasis.

**Methods:**

The “limma” R package was used to analyze three datasets from the Gene Expression Omnibus database (GSE13355, GSE30999 and GSE106992), and the differential genes were screened. The STRING database was used for gene ontology (GO) enrichment analysis, Kyoto encyclopedia of genes and genomes (KEGG) pathway enrichment analysis, and protein–protein interaction network integration. Then, the *IFIT3* subnetwork was extracted and analyzed by gene set enrichment analysis (GSEA) using the Metascape database to verify the effectiveness of gene differentiation and disease tissue identification.

**Results:**

In this study, 426 differential genes were obtained, of which 322 were significantly upregulated and 104 were significantly downregulated. GO enrichment analysis showed that the differential genes were mainly involved in immunity and metabolism; the KEGG pathway enrichment analysis mainly included the chemokine signal pathway, PPAR signal pathway, and IL-17 signal pathway, among others. Based on the *IFIT3* subnetwork analysis, it was found that *IFIT3* was mainly involved in the biological processes of viruses, bacteria, and other microorganisms. The pathways obtained by GSEA were mainly related to immunity, metabolism, and antiviral activities. *IFIT3* was highly expressed in psoriatic lesions and may thus be helpful in the diagnosis of psoriasis.

**Conclusion:**

The differential genes, biological processes, and signal pathways of psoriasis, especially information related to and diagnostic efficiency of the *IFIT3* gene, were obtained by bioinformatics analysis. These results are expected to provide the theoretical basis and new directions for exploring the pathogenesis of psoriasis, in addition to helping with finding diagnostic markers and developing drug treatment targets.

## Introduction

Psoriasis is a chronic inflammatory skin disease characterized by abnormal proliferation of keratinocytes and infiltration of the inflammatory cells. The global incidence of psoriasis in adults is approximately 2%–4% (Chamoun et al., 2015), and psoriasis seriously affects the mental health and quality of life of the patients. In recent years, studies related to psoriasis have mainly focused on hereditary, infection, environmental, immunity, and endocrine conditions. However, the pathogenesis of psoriasis is not clear, and investigations on the molecular mechanisms of psoriasis are still needed. During the acute phase, psoriatic lesions exhibit eczematous changes, making it difficult to distinguish them from atopic dermatitis and posing challenges to the selection of biological agents (Zhou et al., 2024). High-throughput sequencing technology has been widely used to screen disease-related core genes, and high-throughput data analysis and information screening have become important research methods (Li et al., 2014). In the present study, the GeneChip data of psoriasis from the Gene Expression Omnibus (GEO) database were used to identify the differentially expressed genes (DEGs) through bioinformatics methods, and these DEGs were assessed using gene ontology (GO) and Kyoto encyclopedia of genes of genomes (KEGG) pathway enrichment analyses as well as protein–protein interaction (PPI) network integration to determine the hub genes. Hence, this study provides a new method for exploring the molecular mechanisms of psoriasis in depth, mining the potential early diagnostic markers of psoriasis, and exploring drug therapy targets at the genetic level.

## Results

### Screening of DEGs

**FIGURE 1:**
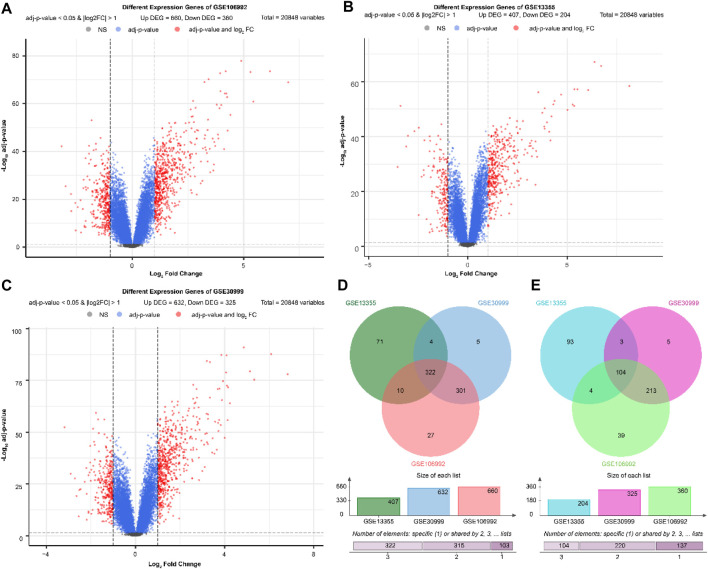
Volcano and bidirectional clustering heat maps of the differential genes in the **(A)** GSE13355, **(B)** GSE30999, and **(C)** GSE106992 datasets. **(D, E)** Cross-analysis of the differential genes in the three datasets.

Volcano maps and bidirectional clustering heat maps (Figure 1) of three datasets from the GEO database (GSE13355, GSE30999 and GSE106992) were obtained according to the standards |log of fold change| > 1 and adj.P.val <0.05. Finally, 426 lesional (LS) vs. non-lesional (NL) differential genes were obtained from cross-analysis screening, of which 322 were significantly upregulated and 104 were significantly downregulated. In the differential gene expression analysis, we employed adjusted *p*-values (from the false discovery rate) to control for the false-positive results arising from multiple comparisons. However, for the subsequent analyses, such as the gene set enrichment analysis (GSEA), we reported the *p*-values directly because the statistical methods of GSEA inherently account for multiple comparisons. The GSEA evaluates significance through the enrichment scores of the gene sets and generates *p*-values based on permutation tests, while also providing adjusted q-values. Furthermore, the results of GSEA focus more on biological significance and overall enrichment of the gene sets rather than differential expressions of individual genes. Therefore, the use of different statistical methods at different stages of analyses is based on the corresponding analytical objectives and characteristics of the methods.

### GO and KEGG pathway enrichment analyses

**FIGURE 2:**
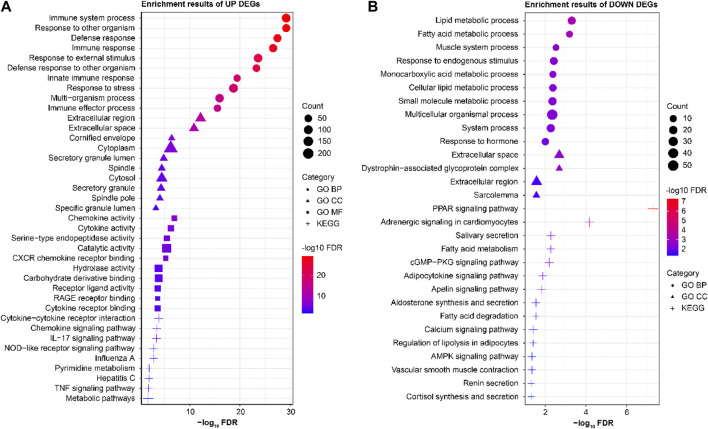
Results of the enrichment analyses of significantly **(A)** upregulated and **(B)** downregulated differential genes.

The STRING database was used to analyze the GO and KEGG enrichments of the 322 upregulated and 104 downregulated differential genes (Figure 2). The GO analysis showed that the upregulated genes were mainly enriched in the cellular components (CCs) (cytoplasm, extracellular space, and cytosol), molecular functions (MFs) (catalytic activity, hydrolase activity, and carbohydrate derivative binding), and biological processes (BPs) (immune responses, responses to external stimuli, and defense responses). The mainly enriched CCs of the downregulated genes were the extracellular spaces, sarcolemma, and dystrophin-associated glycoprotein complex, while the mainly enriched BPs of were lipid metabolism, responses to endogenous stimuli, and multicellular organismal processes. The KEGG enrichment analysis showed that the significantly upregulated differential genes were mainly involved in immune-related functions and pathways, while the significantly downregulated differential genes were mainly involved in metabolic- and signal-transduction-related functions and pathways.

### GSEA of the *IFIT3* subnetwork

Three datasets from the GEO database were analyzed by GSEA, and the significant results were selected for cross-analyses (Table 1). The significant GSEA results from two or more datasets were taken as the GSEA enrichment results for the *IFIT3* gene; thus, 19 positive correlation enrichment results were obtained. Through GSEA, it was concluded that the *IFIT3* gene was mainly related to the immune, metabolic, and antiviral pathways. The high expressions of *IFIT3* are significantly enriched in the antiviral response and immune regulation pathways, suggesting that *IFIT3* may be involved in the occurrence and progression of psoriasis through modulation of these pathways. Furthermore, based on the subnetwork analysis of *IFIT3*, we identified a set of genes that interacted closely with *IFIT3* and are primarily involved in antiviral and antibacterial responses as well as other microbial BPs.

**TABLE 1:** Cross-analysis of GSEA results of the three datasets.

Term	KEGG
Positive	Toll-like receptor signaling pathway
	Cytosolic DNA sensing pathway
	RIGI-like receptor signaling pathway
	Primary immunodeficiency
	Pyrimidine metabolism
	Cell cycle
	NOD-like receptor signaling pathway
	Purine metabolism
	One carbon pool by folate
	DNA replication
	JAK/STAT signaling pathway
	Apoptosis
	Epithelial cell signaling in *Helicobacter Pylori Infection*
	Natural-killer-cell-mediated cytotoxicity
	Cytokine receptor interaction
	Oocyte meiosis
	Amyotrophic lateral sclerosis (ALS)
	Graft-versus-host disease
	Type I diabetes mellitus

### Expression of *IFIT3* and receiver operating characteristics (ROC) analysis of the results

**FIGURE 3:**
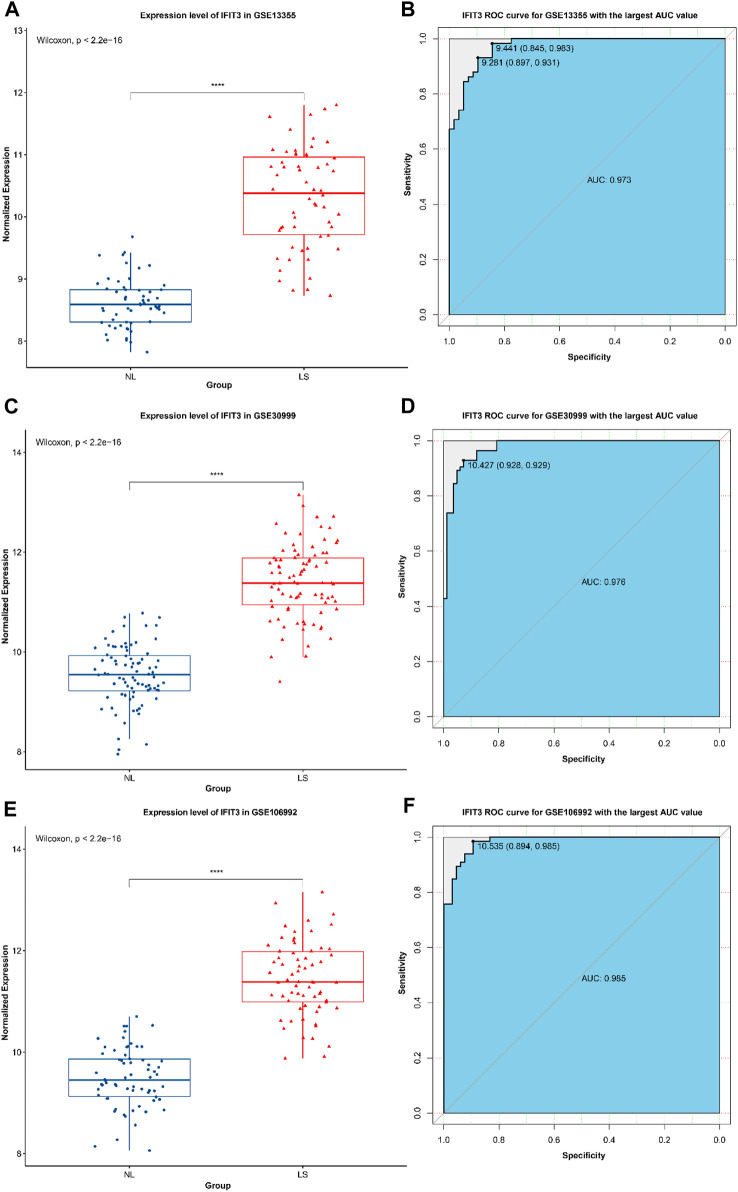
The expression of *IFIT3* in GSE13355 **(A)** and its ROC efficacy in the identification of psoriasis **(B)**. The expression of *IFIT3* in GSE30999 **(C)** and its ROC efficacy in the identification of psoriasis **(D)**. The expression of *IFIT3* in GSE106992 **(E)** and its ROC efficacy in the identification of psoriasis **(F)**.

Box line maps of the three datasets were used to show the expression analyses of *IFIT3* in different groups, and ROC analysis was used to verify the identification efficiency (Figure 3). The results show that the expression levels of *IFIT3* in LS were higher than that in NS in the three datasets, with the area under the curve (AUC) exceeding 0.97, indicating great diagnostic significance.

### Immune infiltration analysis

**FIGURE 4:**
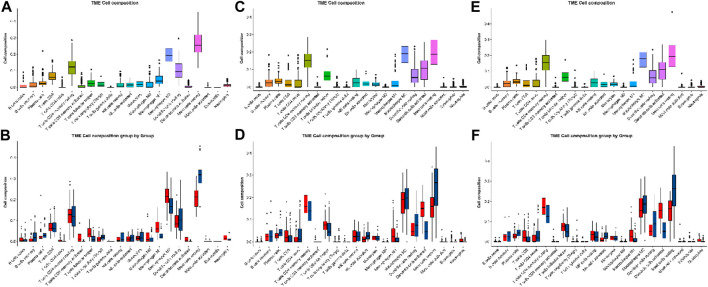
Immune infiltration analyses of the **(A, B)** GSE13355, **(C, D)** GSE30999, and **(E, F)** GSE106992 datasets.

In GSE13355, resting mast cells are the predominant immune cell type, followed by M2 macrophages, resting CD4 memory T cells, and resting dendritic cells. Furthermore, these four main immune cell types exhibit significant differences between the two groups (Figure 4); the resting CD4 memory T cells and M2 macrophages are higher in the LS than NL tissues, whereas the resting mast cells and resting dendritic cells are higher in the NL than LS tissues. In psoriasis, the mast cells express and release IL-17 and IL-22 (Song et al., 2024). In our research, based on analyses of three independent datasets, we have discovered that the primary immune cell types present in the skin tissues of psoriasis patients were resting mast cells, M2 macrophages, and resting CD4 memory T cells. Specifically, the population of the resting mast cells in the LS tissues were significantly lower than that in the NL tissues, whereas the number of resting CD4 memory T cells was markedly elevated in the LS tissues. These observations indicate substantial changes in the mast cells associated with psoriasis and suggest the potential for their functional dysregulation.

## Discussion

With the development of high-throughput chip technologies, bioinformatics methods can be used to screen important genes involved in the pathogenesis of diseases more rapidly, economically, and efficiently, thereby providing a basis for disease diagnosis, treatment, and drug design. The GEO database is a large database established and maintained by the National Biotechnology Information Center of the United States and contains high-throughput data and information uploaded by many research institutions from around the world; however, large portions of these data have not been fully mined (Panahiazar et al., 2017).

In this study, we selected and analyzed the original datasets GSE13355, GSE30999, and GSE106992 from the GEO database, which correspond to the normal control group (non-psoriatic patients), psoriatic NL tissues (psoriatic patients), and psoriatic LS tissues (psoriatic patients). After screening, the samples were included under NL208 and LS207 cases. After standardized data processing, a total of 426 LS vs. NL differential genes were screened, of which 322 were significantly upregulated and 104 were significantly downregulated. Through GO and KEGG enrichment analyses of these differential genes, it was found that the significantly upregulated genes were mainly involved in immune-related functions and pathways, while the significantly downregulated differential genes were mainly involved in metabolism and signal transduction-related functions and pathways. In our enrichment analysis of the psoriasis-related genes, the notably downregulated lipid metabolism and fatty-acid metabolism pathways (Figure 2B) appear to be somewhat inconsistent with the existing understanding that psoriasis is often accompanied by metabolic abnormalities. However, it should be noted that the relationships between psoriasis and metabolic abnormalities are diverse and complex, extending beyond the mere upregulation or downregulation of certain metabolic pathways. For instance, a study utilizing Er-Dong-Xiao-Ke decoction (EDXKD) for the treatment of diabetic-related meibomian gland dysfunction (MGD) (Shi et al., 2024) indicates that activation of the AMPK signaling pathway plays a pivotal role in regulating complex metabolic networks. Specifically, the study demonstrates that EDXKD can modulate lipid and fatty-acid metabolisms by upregulating the AMPK signaling pathway. This suggests that the metabolic abnormalities in psoriasis may involve similar regulatory mechanisms, particularly in the pathways regulated by PPARG and AMPK. Against this research backdrop, downregulation of the lipid and fatty-acid metabolism pathways may not represent simple reductions in the metabolic activity but rather more complex pathophysiological adjustments. For example, this result may reflect cellular rebalancing of energy metabolism under inflammatory conditions to cope with the altered demands of lipids and fatty acids in psoriatic lesions. Furthermore, downregulation of these metabolic pathways may be related to the regulatory pressures exerted by certain drugs or therapeutic approaches on the metabolic pathways, thereby affecting the overall metabolic balance. Thus, our findings offer a novel perspective, revealing the obscure mechanisms in the pathogenesis of psoriasis and encouraging further investigations into the roles of these specific metabolic pathways in the disease. From the PPI analyses of the differential genes, it was found that the *IFIT3* subnetwork was closely related to other groups and that there was a lack of research on the relationships between the *IFIT3* gene and psoriasis; hence the *IFIT3* gene was considered the key research object, and its subnetwork was enriched and analyzed. It was found that the genes in the *IFIT3* subnetwork were mainly involved in the BPs of viruses, bacteria, and other microorganisms. Through the GSEA, it was found that *IFIT3* was mainly related to the immunity, metabolism, and antiviral pathways. Several studies have supported the pivotal roles of *IFIT3* in various immune and inflammatory responses; for instance, the significance of *IFIT3* in the interferon signaling pathway and its function in the antiviral responses induced by viral infections have been widely recognized through extensive research (Yang et al., 2017). The expression level of *IFIT3* in the psoriatic lesions was higher than that in NL tissues and had better identification efficacy, further demonstrating the pertinence of *IFIT3* in the diagnosis and targeted treatment of psoriasis. In addition to *IFIT3*, the highlighted genes include *ISG15*, *MX1*, and *RSAD2*, and members of the OAS family (*OAS1*, *OAS2*, and *OAS3*), warranting further investigations.

The etiology and pathogenesis of psoriasis are complex and cannot be explained by a single factor. At present, the environmental factors related to the occurrence and development of psoriasis include pharyngeal streptococcal infection, emergency events, dry climate, drugs, human immunodeficiency virus (HIV) infection, trauma, smoking, and obesity, among others (Chandran and Raychaudhuri, 2010). The relationship between infection and psoriasis is particularly close; many patients with psoriasis have a history of upper respiratory tract infections before onset or aggravation, especially in guttate psoriasis, children’s psoriasis, and acute attack of psoriasis. Approximately 15% of these cases are caused or aggravated by pharyngeal streptococcal infection (Masood et al., 2000). It has been found that Gram-positive streptococci can activate the innate immune cells through the toll-like receptors TLR2 and TLR4 to participate in the occurrence and development of articular psoriasis (Carrasco et al., 2011). In addition, some scholars have found that the antigenic determinant recognized by T cells in the psoriatic lesions is shared by the streptococcal M protein and keratin; it is also speculated that CD8 + T lymphocytes in the epidermis of psoriatic lesions are the main cells that recognize this antigenic determinant (Valdimarsson et al., 2009). In addition to *Streptococcus*, *Staphylococcus aureus* is involved in the occurrence and development of psoriasis. *S. aureus* exotoxin has super antigenic effects and can directly bind to TCRβ on the surfaces of the T lymphocytes without major histocompatibility complex restrictions to activate them directly. This mechanism is considered to be closely related to the occurrence of psoriasis, especially acute attacks. Moreover, the psoriasis area severity index (PASI) scores were higher for psoriatic patients with cultured *S. aureus* (Balci et al., 2009; Ng et al., 2017). Studies have shown that in patients with psoriasis, *Malassezia* can be isolated from skin lesions, which may be related to psoriatic scale loosening and long-term topical use of glucocorticoids (Akaza et al., 2012). *Malassezia* may stimulate human epidermal keratinocytes to secrete inflammatory cytokines, leading to differentiation of Th cell subsets and participation in the occurrence and development of psoriasis (Nyfors et al., 1976). As early as 1985, some scholars had reported that HIV could lead to the occurrence and aggravation of psoriasis (Johnson et al., 1985). When the CD4^+^ cell count was less than 450 cells/mm^3^, psoriasis patients infected with 80% HIV were in the progressive stage; when the CD4^+^ cell count was less than 200 cells/mm^3^, the risk of psoriasis exacerbation was nine times higher (Alpalhão et al., 2019); both these conditions were associated with TNF-α and IFN-γ (Craven et al., 2001). The HIVnef protein can be used as a superantigen to affect psoriasis, while the HIVtat protein can directly stimulate the proliferation of epidermal and dermal cells (Ceccarelli et al., 2019). The above studies suggested that bacteria, viruses, and other infectious factors may induce or aggravate psoriasis through the toll-like receptors, IFN-related signaling pathways, and T cell activation, which are consistent with our findings.

The members of the IFIT family were discovered early for the interferon-stimulating genes (ISGs) located on chromosome 10 in the human genome, and a total of four members were found: IFIT1/ISG56, IFIT2/ISG54, IFIT3/ISG60, and IFIT5/58 (Xiang et al., 2013). Among these, *IFIT3* is an important host antiviral immune effector molecule that inhibits the replication of many kinds of DNA and RNA viruses (Hsu et al., 2013), promotes IFN-induced apoptosis *in vivo* and *in vitro* (Ying-yun et al., 2017), positively regulates the RIG-1 pathway by promoting interactions between STING and TBK1, enhances the activation of IRF3 and NF-κB pathways, promotes the production of IFN-1, and enhances the antiviral effect of cells. *IFIT3* induces the IFN signaling pathway and represses adenoviral immediate early gene expression; it has also been reported that *IFIT3* can enhance the anti‐hepatitis B virus effects of IFN-α. The replication of many other viruses is inhibited by *IFIT3* with unknown mechanisms; *IFIT3*-inhibited viral replication is usually correlated with the expressions of a subset of immune molecules. However, *IFIT3* can also exert a direct inhibitory effect on the replications of partial viruses, and the specific mechanisms deserve in-depth exploration (Zhang et al., 2023). Therefore, *IFIT3* may interfere with psoriasis by related antivirus regulating pathways and induce the production of IFN. In this study, the *IFIT3* gene was analyzed through bioinformatics, and it was found that *IFIT3* was closely related to psoriasis-related infection and immune pathways as well as cytokines; it showed high expressions in psoriatic lesions and had good identification value. These results have a certain guiding significance for follow-up research.

To summarize, using various bioinformatics methods, this study discusses the possible mechanisms of occurrence and development of psoriasis from different perspectives along with deep data mining to identify the key gene, namely *IFIT3*. Our research not only uncovers the pivotal roles of the *IFIT3* gene in the immune and signal transduction pathways but also identifies its principal associated pathways in psoriasis through GSEA. More importantly, we substantiated the efficacy of the *IFIT3* gene as a diagnostic marker for psoriasis via ROC curve analysis, with the AUC values consistently exceeding 0.97 across the three datasets and demonstrating the exceptionally high diagnostic efficacy. Furthermore, detailed analyses of immune cell infiltration in the tissues of psoriasis patients revealed significant differences between LS and NL tissues in terms of the key immune cell types, thereby providing additional support for the important functions of *IFIT3* in the immune responses to psoriasis. The functional and pathway enrichment analyses of the *IFIT3* gene help in further understanding its function in psoriasis. However, this study is based on the analysis and prediction of existing data in the GEO database and lacks experimental verification; the single-group studies do not provide comprehensive understanding of the gene functions, and there may be false-negative or false-positive conclusions. The *IFIT3* gene functions can be verified by subsequent large samples to further identify highly sensitive and specific diagnostic markers as well as provide strong support for the targeted treatment of psoriasis-related diseases.

## Conclusion

The present study identifies multiple DEGs associated with psoriasis and highlights the roles of *IFIT3* while providing directions for further research on the development and immune mechanisms of psoriasis. Thus, we explore the pathogenesis of psoriasis in depth and propose potential target genes for psoriatic therapy.

## Materials and methods

### Data acquisition of the gene expression chips

The gene expression datasets analyzed in this study were GSE13355, GSE30999, and GSE106992 obtained from the GEO database. Among these, the GSE13355 dataset included normal control groups (non-psoriatic patients), psoriatic NL tissues (psoriatic patients), and psoriatic LS tissues (psoriatic patients). The GSE30999 and GSE106992 datasets both included psoriatic NL as well as LS tissues (psoriatic patients). The analyses in the present study were based on these three datasets for the molecular mechanisms of LS and NL tissues in patients with psoriasis without drug treatment. The GSE13355 dataset contains 180 samples, but the actual analysis contains 116 psoriasis samples after excluding 64 normal samples. Both the GSE30999 and GSE106992 datasets contain psoriasis LS and NL information from patients who have not received drug treatment. Owing to the lack of control groups in the GSE30999 and GSE106992 datasets, we mainly focused on the molecular mechanisms of the psoriasis patients.

### Data standardization

**FIGURE 5:**
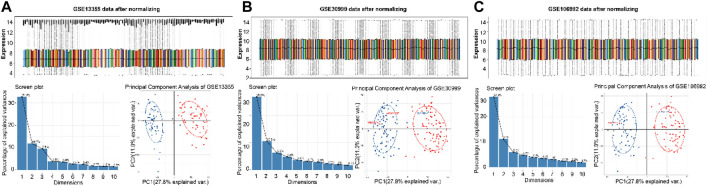
Results of dimensional test and PCA analysis of GSE13355 **(A)**, GSE30999 **(B)** and GSE106992 **(C)**.

After downloading the original data, we used R software and its “affy” package for data reading and standardized the data through the robust multiarray average (RMA) method to ensure consistency and comparability. Then, we reannotated the chip using the annotation file of the corresponding platform GPL570. The probe without gene symbols and deletion of multiple gene symbols corresponding to one probe were implemented, and the maximum value of the single gene symbol corresponding to multiple probes was taken as the gene expression value for follow-up analyses. Dimensional tests and principal component analysis (PCA) were performed on the expression profiles, and the samples with obvious outliers in the results of PCA were deleted while the differences of the remaining samples were analyzed. The results of the PCA showed that three samples in the GSE30999 dataset (GSM768074, GSM768097, and GSM768135) were obvious outliers while two samples in GSE106992 (GSM2859108 and GSM2859086) showed obvious advantages, so these five samples were deleted. Thus, a total of 116 samples were used from GSM13355, including 58 NL and 58 LS samples; 167 samples were used from GSM30999, including 84 NL and 83 LS samples; 132 eligible untreated samples were used from GSM106992, including 66 NL and 66 LS samples (Figure 5).

### Differential gene screening

The differential gene analysis was carried out using the “limma” package in R (Ritchie et al., 2015). The volcano and bidirectional clustering heat maps were drawn and visualized with the thresholds |log of fold change| > 1 and adj.P.val < 0.05. Following the differential analyses of the three datasets, jveen (Panahiazar et al., 2017) was used to cross-analyze the genes with significant differences in the three datasets. The significantly upregulated genes in all three datasets were regarded as LS vs. significantly upregulated differential genes as NL, and the significantly downregulated genes in all three datasets were regarded as LS vs. significantly downregulated differential genes as NL.

### GO and KEGG pathway enrichment analyses

The STRING database (https://string-db.org) was used to analyze the enrichments of the significantly upregulated and downregulated differential genes for the GO and KEGG pathways, respectively. The top-ten results of each item were visualized using air bubble diagrams.

### PPI network construction and analysis

Using the STRING database for PPI analysis of the differential genes, the minimum required interaction score was set to 0.7 (high confidence), while the other parameters were set to the default values of the STRING database. The “Igraph” R package (Csardi and Nepusz, 2006) was used to visualize the PPI network, and the cluster fast greedy method was used to divide the PPI network into modules.

### Enrichment analyses of submodules containing *IFIT3*

**FIGURE 6:**
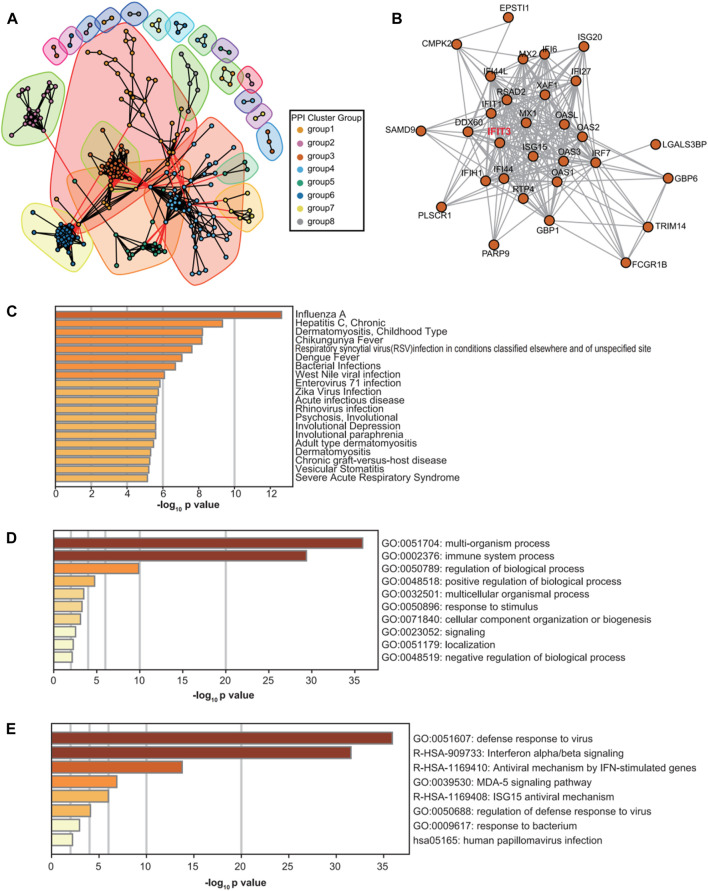
PPI network diagram and module diagram **(A)**. Subnetwork diagram of *IFIT3* on the PPI network **(B)**. Metascape results of enrichment analysis of *IFIT3* subnetwork genes based on DisGeNET disease database **(C)**. Results of enrichment analysis of IFIT3 subnetwork genes in Metascape database **(D, E)**.

Once the PPI network was divided, the modules containing the *IFIT3* gene were retrieved, and the genes contained in these modules were uploaded to the Metascape database (Yingyao et al., 2019) for correlation and related functions analyses (Figure 6). The above results showed that the *IFIT3* gene modules were mainly involved in viral- and bacterial-infection-related diseases.

### GSEA

GSEA is an enrichment algorithm used by scientists at the Broad Institute. In this study, the results of the enrichment analyses of the submodules were used to perform cross-analysis with the jveen tool. The significant results supported by at least two of the three datasets (*p* < 0.05) were taken as the results of the GSEA of *IFIT3*.

### Expression of *IFIT3* and ROC analysis

Box diagrams (Kassambara, 2020) were used to show the expression groupings of *IFIT3* in the three datasets, and the ROC curve (Robin et al., 2011) was used to verify the diagnostic identification efficiency of the genes.

### Estimation of immune cell infiltration

To investigate the cell fractions based on the tissue gene expression profiles in the risk groups, CIBERSORT was employed to compute the proportions of 22 types of immune cells in all samples. The Wilcoxon test was employed to screen for significantly different immune cells; then, Spearman’s method was employed to calculate the relationship between each signature and the different immune cells. The R package “ggplot2” was used to visualize the data via bar charts, correlation heat maps, heat maps, and violin plots.

### Statistical analyses

All statistical analyses were performed using R version 4.1.2, and the statistical tests were considered statistically significant at *p* < 0.05; all estimates were considered to be significant at a confidence interval of 95%.

## Data Availability

The original contributions presented in the study are included in the article/Supplementary Material, and any further inquiries may be directed to the corresponding author.

## Abbreviations

GEO: Gene Expression Omnibus; DEGs: differentially expressed genes; GO: gene ontology; KEGG: Kyoto encyclopedia of genes and genomes; PPI: protein–protein interaction; IFIT3: interferon-induced protein with tetratricopeptide repeats 3; GSEA: gene set enrichment analysis; LS: lesional; NL: non-lesional; CC: cellular component; MF: molecular function; BP: biological process; ROC: receiver operating characteristics; AUC: area under the curve; EDXKD: Er-Dong-Xiao-Ke decoction; MGD: meibomian gland dysfunction; PASI: psoriasis area severity index; HIV: human immunodeficiency virus; ISGs: interferon-stimulating genes; RMA: robust multiarray average; PCA: principal component analysis.
