# Supplementary figures and images for: Unraveling the roles of IFIT3 gene and immune-metabolic pathways in psoriasis: a bioinformatics exploration for diagnostic markers and therapeutic targets
Source: Front Mol Biosci. 2024 Aug 22;11:1439837. doi: 10.3389/fmolb.2024.1439837 (PMC11374644; doi:10.3389/fmolb.2024.1439837)

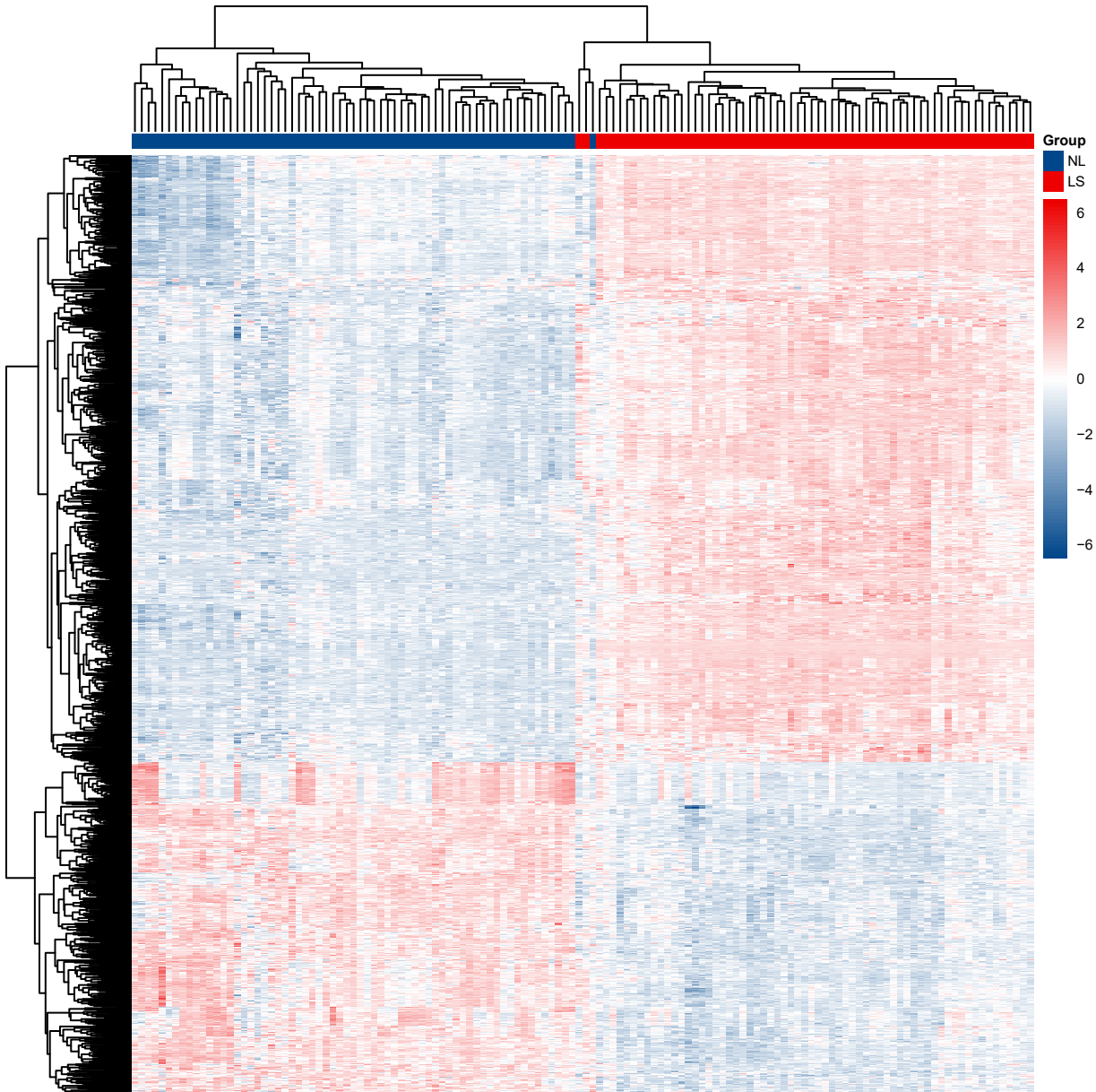

Supplement: Supplementary file 1 [file Image2.pdf]

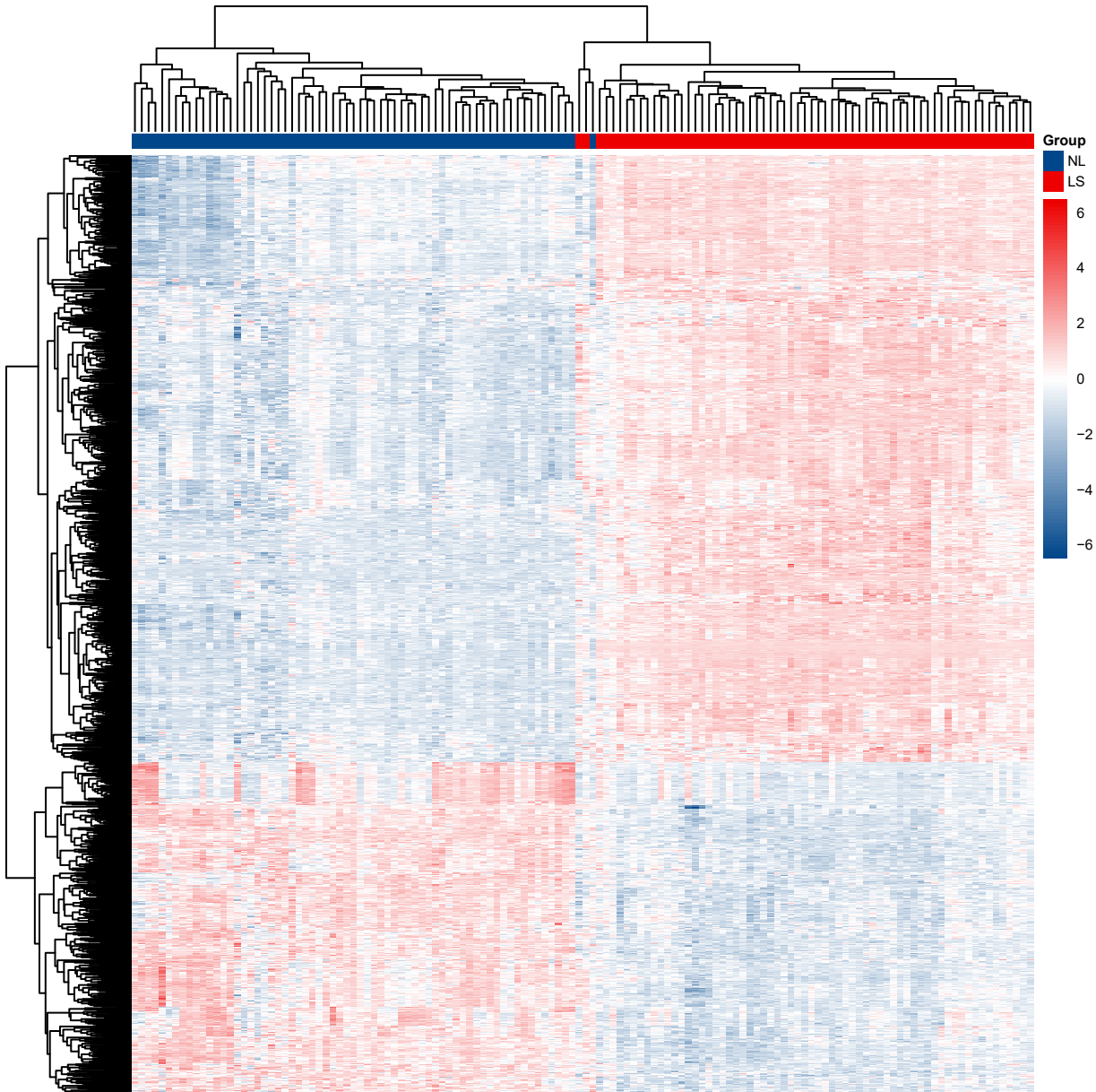

Supplement: Supplementary file 2 [file Image1.pdf]
